# Mortality from cervical cancer in Brazil: an ecological epidemiologic study of a 22-year analysis

**DOI:** 10.3332/ecancer.2020.1064

**Published:** 2020-06-25

**Authors:** Diego Bessa Dantas, Thalita da Luz Costa, Amanda Suzane Alves da Silva, Fabiana de Campos Gomes, João Simão de Melo-Neto

**Affiliations:** 1 Federal University of Pará (UFPA). Belém, Pará, Brazil; 2 School of Medicine of São José do Rio Preto (FAMERP), São José do Rio Preto, SP, Brazil

**Keywords:** Brazil, uterine neoplasms, mortality, epidemiology

## Abstract

Mortality data obtained from the mortality information system (SIM) identified a total of 103,094 women with cervical cancer in Brazil. However, associations between mortality and sociodemographic variables in these patients are not fully understood. Therefore, this study aimed to analyse the sociodemographic factors (geographic region, age, race and marital status) that predict cervical cancer mortality in Brazil between 1996 and 2017. A descriptive, analytic and retrospective study was carried out using secondary data on deaths from cervical cancer recorded in the SIM-DATASUS. Deaths reported between 1996 and 2017 in the health information system and classified by the International Classification of Diseases-10 were included. Sociodemographic factors (geographic regions, age, sex and race) were subjected to inferential analysis for a relation with mortality. Mortality increases during the aging process after the third decade of life. However, single women who die are usually diagnosed with cancer in the early stage of the disease. The mortality rate is higher in Black women and women living in the North, South and Southeast regions of Brazil. Yellow women have a lower mortality in the country. Besides, each region has specific characteristics in relation to race and marital status. White women who died had some form of stable union during life, whereas the other races were more associated with single marital status. Thus, the sociodemographic factors that predict mortality in women with cervical cancer in Brazil were identified and can be used to guide the public health policies.

## Introduction

Cervical cancer is the fourth most commonly diagnosed cancer amongst women worldwide. It is estimated that 90% of deaths from this disease are recorded in low- and middle-income countries. Morbidity and mortality from cervical cancer are much lower in developed countries because of the availability of efficient and affordable screening programs as well as diagnostic and treatment facilities. Meanwhile, in the low- and middle-income countries, the low survival rate is attributable to late diagnoses and failure to receive or complete the prescribed treatment regimens amongst patients [1].

Human papillomavirus (HPV) is the leading cause of cervical cancer worldwide [2]. However, other specific factors have been found to increase the risk of progression to cancer amongst infected women. These include age, education, income, immigrant status, acculturation, cultural beliefs about modesty and sexual behaviour, family-centred values and existing social networks [3].

Cervical cancer is the most common pelvic cancer in Brazil and is the second most prevalent cancer in women after breast cancer. It is the fourth leading cause of cancer mortality in the country [4].

Many factors have been discussed as barriers to health service access in Brazil, namely, low socioeconomic status and inadequate coverage of cervical cancer screening [5]. The comparative studies have found associations between mortality prediction, survival and sociodemographic variables in cancer patients, reinforcing the need to increase the number of studies to generate a consistent body of literature [6].

There are no consistent Brazilian studies demonstrating an association of these factors with the possibility of death from cervical cancer. This study aimed to analyse the sociodemographic factors that predict cervical cancer mortality in Brazil. Specifically, the relation of the following sociodemographic factors between 1996 and 2017 will be evaluated: geographic region, age, race and mortality.

## Methods

### Ethics

This study analysed the secondary data available in DATASUS [7]. The data are public with unrestricted access and use. An ethical evaluation by the Research Ethics Committee was not required according to the guidelines of the National Council of Health, No. 510 (April 07, 2016).

### Type of study

This was an ecological study analysing the data on deaths from cervical cancer recorded in the mortality information system (SIM) of the Ministry of Health of Brazil.

### Database

SIM is a secondary database available at the Informatics Department of the Brazilian National Health System (DATASUS) of the Ministry of Health [7]. The reported deaths from 1996 to 2017 in Brazil, which were recorded in the health information system (TABNET), classified by the International Classification of Diseases-10 (ICD-10) [8] and defined according to the 10th revision code C53, were included. Dates outside of the proposed period of study were excluded. Data from the total female population (1996–2017) for age–period–cohort analysis (APC) and specific population (2001–2017), according to geographic region and race to verify the mortality rate, were obtained from the SIDRA-IBGE platform [9].

### Study variables

The sociodemographic factors studied were geographic region (North, Northeast, Midwest, South or Southeast), race (Brown, White, Black, Yellow or Indigenous), age (19 or less, 20–29, 30–39, 40–49, 50–59, 60–69, 70–79 or 80 years or more) and marital status (single, married, widowed or divorced).

### Statistical analysis

The data were subjected to the descriptive and inferential analyses. The relative and absolute frequencies were used for data description.

The APC obtained by parameters estimated using the APC Web Tool (Biostatistics Branch, National Cancer Institute, Bethesda, MD, USA) [10] was used. The appropriate model was applied, which explains the identification problem to determine the variations in mortality due to the independent effects of age groups, death calendar periods and birth cohorts. For all variables analysed in this study, the following functions were estimated: net deviation (global annual percentage change according to the calendar period and the birth cohort), local deviations (annual percentage changes for each age group according to the calendar period and birth cohort), all age deviations (the adjusted longitudinal and transverse age curves are log-linear), all period deviations (adjusted time trends and period rates are log-linear), all cohort deviations (cohort rates are log-linear, and all local deviations are equal to net deviations) and all period rate (or cohort) (RR) ratios (age incidence pattern in each period [or cohort]). The Wald test was used to verify the significant difference, with *p* < 0.05 being considered to be statistically significant.

To verify the differences between the mortality rate, the data were subjected to the Kolmogorov–Smirnov normality test. The results are presented as median and dispersion variables through graphs, as appropriate for non-parametric tests. The Scheirer–Ray–Hare test with the *post hoc* Mann–Whitney U-test was applied to assess the group heterogeneity in geographic region and race.

The Chi-squared test was used to examine associations between cervical cancer mortality and marital status. Odds ratios (ORs) with 95% confidence intervals (95% CI) were used to quantify the degree of association. The mortality rate was not calculated for marital status due to the lack of these data available over the period at the SIDRA-IBGE platform [9].

## Results

### Age–period–cohort

**Figure 1.**
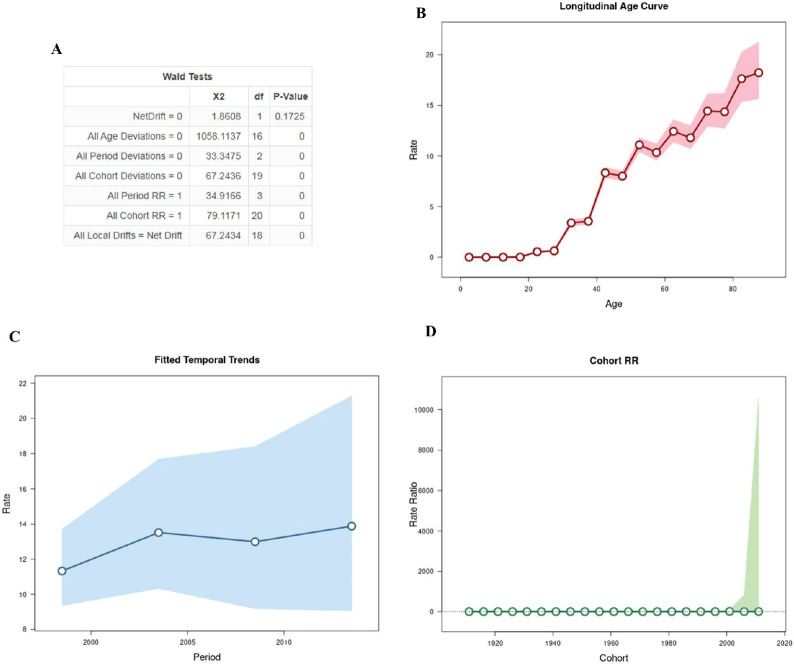
Age–period–cohort analysis with the Wald test, representation of the mortality rate by age (B), period (C) and birth cohort (D).

After analysing the APC, the results are shown in Figure 1. The annual percentage change in expected age-adjusted rates obtained through the net drift was not significant. In relation to age, the analysis demonstrated that there is a greater risk of progressing to death with advancing age after 32.5 years (rate = 3.387 [95% CI, 3.055–3.755]), with a greater peak in individuals aged 80 years (Figure 1B). On the contrary, younger women aged 27.5 years had a lower risk than older women (rate = 0.626 [95% CI, 0.527–0.743]). Regarding the period, the results demonstrated that the rate periods are log-linear, resulting in an increase of mortality in recent years (Figure 1C). Figure 1D shows a pattern of age incidence in all birth cohorts.

### Geographic region and race

**Figure 2.**
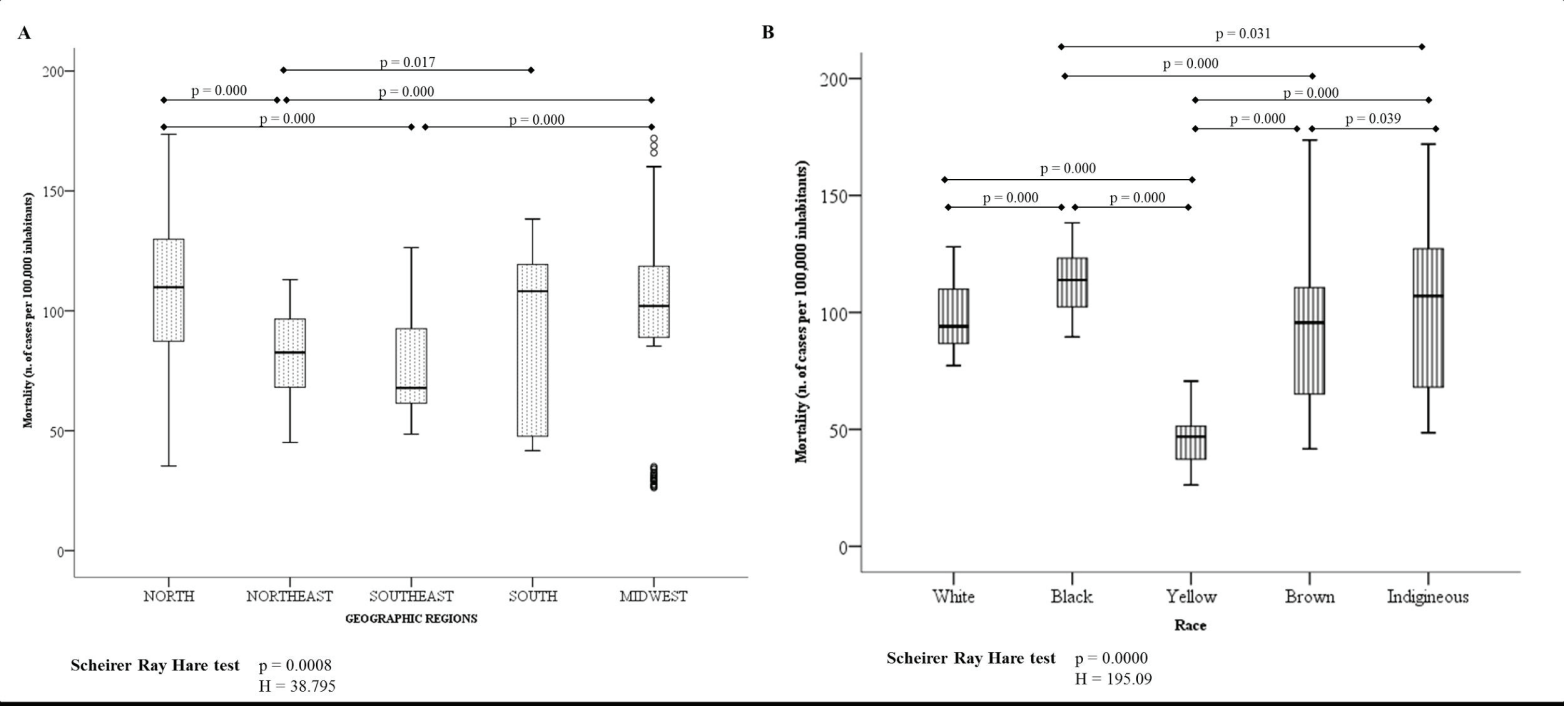
Mortality rate for every 100,000 inhabitants according to geographic region (A) and race (B).

Figure 2A shows that the mortality rate for every 100,000 inhabitants is greater in the geographic region: North, South and Midwest. In relation to race, Black and Yellow women had the highest and lowest mortality rate, respectively (Figure 2B).

**Figure 3.**
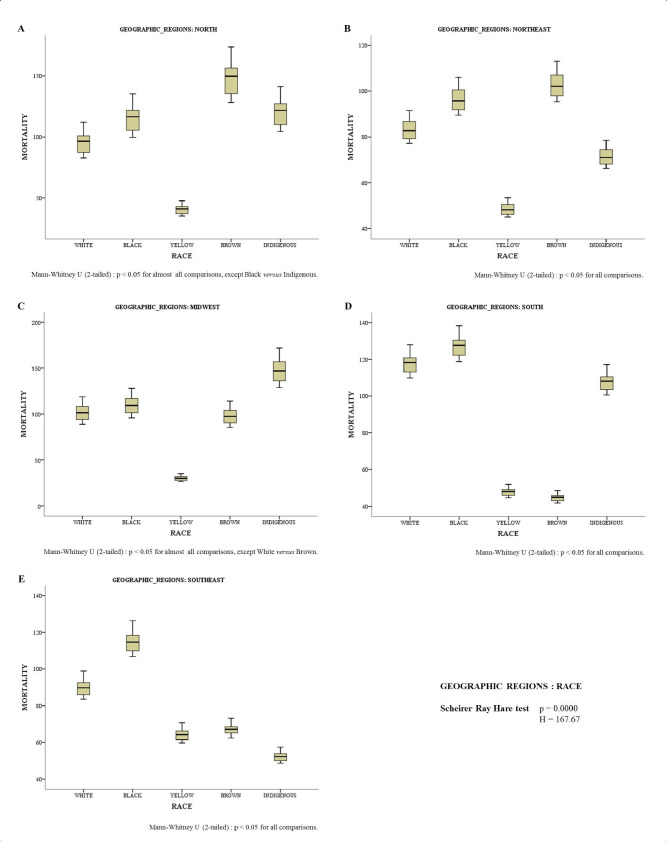
Mortality rate for every 100,000 inhabitants according to race in different geographic regions: North (A), Northeast (B), Midwest (C), South (D) and Southeast (E).

Figure 3 shows the differences between races in each demographic region. The mortality rate was higher in Brown women in the North (Figure 3A) and Northeast (Figure 3B) regions. In the Midwest (Figure 3C) region, the results showed that the mortality rate from cervical cancer is proportionally higher in Indigenous women. In the South (Figure 3D) and Southeast (Figure 3E) regions, mortality was higher in Black women.

Yellow women had a lower mortality rate in the North, Northeast and Midwest regions (Figure 3A–C). Brown and Indigenous women had a lower mortality rate in the South and Southeast regions (Figure 3D and E, respectively).

### Marital status versus geographic regions, race and age group

Table 1 shows the association between marital status and geographic regions, race and age related to cervical cancer mortality. With respect to marital status, single status in the North and Northeast, married and widowed status in the South and divorced status in the South, Southeast and Midwest regions were all highly associated with cervical cancer mortality. Married, widowed or divorced White women; single Black, Brown and Indigenous women and widowed Yellow women showed a strong association with a higher mortality. Besides, <19–49-year-old single women, 40–69-year-old married and divorced women and widowed women with over 60 year-old also had an increased odds of mortality.

## Discussion

Cervical cancer is one of the most prevalent types of cancer in women worldwide [1], and understanding the association between mortality and sociodemographic variables in Brazilian patients is considerably important. In this context, this study analysed whether the factors such as age, geographic region, race and marital status predict mortality in a 22-year period. In this study, we found that these sociodemographic factors present specific characteristics that predict mortality in these patients.

In this study, we observed a high rate of mortality after 32.5 years of age, with a peak in elderly women. The biological and aetiological differences or the presence of risk factors between age groups have been pointed out as the determining factors [11, 12]. The aetiological factor HPV is one of the main influences of biological characteristics, being considered as a determining factor for invasive cancer [13]. Besides, the physiological changes resulting from aging in the cervix may alter HPV oncogenic subtypes in older women [14]. However, the studies [15, 16] in the Asian population indicate that age does not interfere with prognosis in women with cervical cancer. In this context, in this study, we emphasise that Yellow women had the lowest mortality rate in Brazil.

Furthermore, we observed that single women aged over 19 years are associated with high mortality. The presence of mortality in this early age group has been observed in other studies, which point to the histopathological evolution of adenocarcinoma [17, 18], and has been associated with the most prevalent HPV subtypes in younger women (HPV-18 and HPV-45) [19]. This type of cancer has a worse prognosis compared to squamous cell carcinoma, with accelerated tumour progression that may not be detected early [20–23].

Regarding race, we found that Black women have the highest mortality rate in Brazil. The previous studies [24, 25] have shown that this population has a higher risk of mortality. This result may be related to some factors, such as access to screening tests, staging at the time of diagnosis, quality of healthcare services, treatment inequalities and cultural and social factors [24, 25].

The mortality rate was higher in women living in the North, South and Midwest regions of Brazil. According to Vale et al. [26], regional differences are associated with the level of development and socioeconomic status of women, which may be due to inequalities in healthcare access and difficulties with planning preventive and therapeutic actions.

Furthermore, several barriers to access have been described as limiting healthcare services for Indigenous people in different regions worldwide [27, 28]. Similar to the previous studies, in the present study, we can verify that the highest mortality rate was observed in the Indigenous population in the Midwest region. The quality of cervical cancer screening in self-declared Indigenous women has been marked with a higher prevalence of three negative points: lack of access to the service, Papanicolaou smear performed more than 3 years ago and lack of guidance on the examination [29]. Although the Midwest region is the second most urbanised region in the country, the accelerated growth has led to a rapid concentration of slums, disorganised regions, poor infrastructure and the low concentration of Indigenous people in urban areas [30]. Besides, we found high mortality rates associated with Browns in the North and Northeast regions, being compatible with a similar pattern in performing the Papanicolaou smear from healthcare services in both regions [31, 32].

Regarding marital status, the region analysed the presented specific characteristics. Marital status may influence the quality of life, health status and survival of patients [33]. Single marital status is associated with a worse prognosis and increased the risk of mortality in women with gynaecological neoplasms compared to married women [33]. These results are similar to those found in this study, referring to the North and Northeast regions. Besides, divorced marital status was more associated with mortality in the South, Southeast and Midwest regions. A social support is a factor that contributes to married women having better psychosocial and general health because they adopt healthier habits, perform physical activity and pay more attention to their health, performing practices of prevention, screening and treatment of the disease [33, 34]. However, a separation process can cause a change in these behaviours.

In this study, White women who died had some form of stable union during life, whereas other races are more associated with single marital status. A previous study [35] showed that there is a high prevalence of White women being married at the time of diagnosis and Black being unmarried (single, divorced and widowed). Unmarried women have lower rates of cervical cancer screening than married women [35]. The number of single women is increasing; thus, early detection should be a priority for this population. Thus, factors such as improvement of healthcare services, preventive and detection tests, access to services and improvement of quality of care should be considered [35].

To determine the sociodemographic factors that may predict cervical cancer mortality in Brazil, similarly to other developing countries, there are limitations in the quality of data collection with a large number of unknown or unreported data that significantly impair several studies using secondary banks [36]. Finally, in this study, we did not analyse the mortality rate before 2001 and according to marital status because the national databases do not offer data from the female population, regardless of the presence of cancer.

## Conclusions

Mortality increases during the aging process after the third decade of life. However, single women who died are usually diagnosed with cancer in the early stage of the disease. The mortality rate is higher in Black women and women living in the North, South and Midwest regions of Brazil. Yellow women have a lower mortality in the country. Besides, each region has specific characteristics in relation to race and marital status. White women who died had some form of stable union during life, whereas the other races are more associated with single marital status.

Thus, in this study, we found that the sociodemographic factors such as age, geographic regions, race and marital status present specific characteristics that predict cervical cancer mortality in Brazil. These findings can be used to direct the new guidelines and improve the guide of public health policies.

## List of abbreviations

HPV: Human papillomavirus
SIM : Mortality information system
OR: Odds ratio
CI: Confidence intervals
NHSS: National household sample survey

## Ethics approval and consent to participate

This research does not require the ethics committee approval because it is an epidemiological study that analyses the secondary data available in DATASUS. The data are public with unrestricted access and use.

## Consent for publication

Not applicable.

## Availability of data and materials

The datasets generated and/or analysed during the present study are already public and available in DATASUS. Still, the collected data are available via the corresponding author. These data sets are included in the supplemental information files in this submission.

## Conflicts of interest

The authors declare that they have no conflict of interest.

## Funding

This research was not funded.

## Authors’ contributions

All authors had substantial contributions to the conception, study design, data interpretation and writing of the work. TLC and JSMN were major contributors to the writing of the manuscript. JSMN and FCG reviewed the work substantively. All authors read and approved the final manuscript.

## Figures and Tables

**Table 1.** Association of marital status with geographic region, race and age group.

Marital status					
	Single	Married	Widowed	Divorced	Others*
Geographic regions					
North	*N* = 4234 *p* = 0.0001 OR =1.522 95% CI: 1.458–1.587	*N* =3700 *p* = 0.1969 OR = 1.029 95% CI: 0.985–1.074	*N* = 1655 *p* = 0.0001 OR = 0.638 95% CI: 0.604–0.674	*N* = 285 *p* = 0.0001 OR = 0.451 95% CI: 0.399–0.509	*N* = 1278
Northeast	*N* = 10169 *p* = 0.0001 OR = 1.421 95% CI: 1.379–1.464	*N* = 9690 *p* = 0.1982 OR = 1.019 95% CI: 0.990–1.050	*N* = 5404 *p* = 0.0001 OR = 0.821 95% CI: 0.793–0.850	*N* = 783 *p* = 0.0001 OR = 0.119 95% CI: 0.110–0.128	*N* = 3388
Southeast	*N* = 11485 *p* = 0.0001 OR = 0.847 95% CI: 0.823–0.871	*N* = 12509 *p* = 0.0001 OR =0.853 95% CI: 0.830–0.877	*N* = 9445 *p* = 0.0001 OR = 0.886 95% CI: 0.856–0.916	*N* = 2695 P = 0.0001 OR = 1.589 95% CI: 1.505–1.679	*N* = 2229
South	*N* = 3876 *p* = 0.0001 OR = 0.691 95% CI: 0.664–0.719	*N* = 6271 *p* = 0.0001 OR = 1.250 95% CI: 1.207–1.295	*N* = 3826 *p* = 0.0001 OR = 1.138 95% CI: 1.093–1.184	*N* = 1205 *p* = 0.0001 OR = 15.020 95% CI: 13.824–16.320	*N* = 1101
Midwest	*N* = 2328 *p* =0.197 OR = 0.966 95% CI: 0.917–1.017	*N* = 2570 *p* = 0.9317 OR = 0.997 95% CI: 0.948–1.049	*N* = 1553 *p* = 0.0459 OR = 0.941 95% CI: 0.888–0.998	*N* = 529 *p* = 0.0001 OR = 1.357 95% CI: 1.237–1.490	*N* = 886
Race					
White	*N* = 11673 *p* = 0.0001 OR = 0.539 95% CI: 0.524–0.555	*N* = 16238 *p* = 0.0001 OR = 1.208 95% CI: 1.174–1.242	*N* = 10984 *p* = 0.0001 OR = 1.393 95% CI: 1.348–1.438	*N* = 3321 *p* = 0.0001 OR = 1.915 95% CI: 1.805–2.032	*N* = 2560
Black	*N* = 2989 *p* = 0.0001 OR = 1.502 95% CI: 1.429–1.579	*N* = 2044 *p* = 0.0001 OR = 0.720 95% CI: 0.683–0.760	*N* = 1569 *p* = 0.4763 OR = 0.978 95% CI: 0.922–1.038	*N* = 295 *p* = 0.0001 OR = 0.672 95% CI: 0.595–0.757	*N* = 580
Yellow	*N* = 144 *p* = 0.0011 OR = 0.722 95% CI: 0.596–0.876	*N* = 205 *p* = 0.1586 OR = 1.140 95% CI: 0.955–1.360	*N* = 153 *p* = 0.0008 OR =1.387 95% CI: 1.148–1.676	*N* = 19 *p* = 0.0254 OR = 0.583 95% CI: 0.368–0.923	*N* = 35
Brown	*N* = 14086 *p* = 0.0001 OR = 1.657 95% CI: 1.610–1.706	*N* = 11914 *p* = 0.0001 OR = 0.904 95% CI: 0.878–0.930	*N* = 6655 *p* = 0.0001 OR = 0.705 95% CI: 0.681–0.729	*N* = 1468 *p* = 0.0001 OR = 0.572 95% CI: 0.537–0.608	*N* = 3362
Indigenous	*N* = 146 *p* = 0.0004 OR = 1.483 95% CI: 1.193–1.842	*N* = 116 *p* = 0.6195 OR =0.938 95% CI: 0.748–1.176	*N* = 66 *p* = 0.1742 OR = 0.822 95% CI: 0.627–1.077	*N* = 5 *p* = 0.0007 OR = 0.235 95% CI: 0.097– 0.568	*N* = 76
Others*	*N* = 2172	*N* = 2874	*N* = 1703	*N* = 288	*N* = 2072
Age					
≤19 years	*N* = 45 *p* = 0.0001 OR = 7.207 95% CI: 3.812–13.627	*N* = 09 *p* = 0.0017 OR = 0.324 95% CI: 0.159–0.660	*N* = 02 *p* = 0.0008 OR =0.120 95% CI: 0.029–0.493	*N* = 01 *p* = 0.2925 OR = 0.283 95% CI: 0.039–2.049	*N* = 09
20–29 years	*N* = 1805 *p* = 0.0001 OR = 6.402 95% CI: 5.820–7.042	*N* = 517 *p* = 0.0001 OR = 0.473 95% CI: 0.429–0.522	*N* = 23 *p* = 0.0001 OR = 0.031 95% CI: 0.020–0.047	*N* = 29 *p* = 0.0001 OR = 0.192 95% CI: 0.133–0.277	*N* = 294
30–39 years	*N* = 6313 *p* = 0.0001 OR = 3.499 95% CI: 3.354–3.650	*N* = 3431 *p* = 0.0001 OR = 0.839 95% CI: 0.804–0.876	*N* = 215 *p* = 0.0001 OR = 0.060 95% CI: 0.052–0.069	*N* = 402 *p* = 0.0001 OR = 0.612 95% CI: 0.552–0.679	*N* = 1454
40–49 years	*N* = 8459 *p* = 0.0001 OR = 1.727 95% CI: 1.671–1.784	*N* = 8042 *p* = 0.0001 OR = 1.641 95% CI: 1.588–1.696	*N* = 1233 *p* = 0.0001 OR = 0.1811 95% CI: 0.170–0.192	*N* = 1339 *p* = 0.0001 OR = 1.265 95% CI: 1.187–1.349	*N* = 2168
50–59 years	*N* = 6483 *p* = 0.0001 OR = 0.831 95% CI: 0.804–0.859	*N* = 9393 *p* = 0.0001 OR = 1.581 95% CI: 1.532–1.631	*N* = 3244 *p* = 0.0001 OR = 0.539 95% CI: 0.517–0.562	*N* = 1730 *p* = 0.0001 OR = 1.644 95% CI: 1.549–1.744	*N* = 1893
60–69 years	*N* = 4177 *p* = 0.0001 OR = 0.527 95% CI: 0.507–0.547	*N* = 7097 *p* = 0.0001 OR = 1.192 95% CI: 1.152–1.232	*N* = 5299 *p* = 0.0001 OR =1.545 95% CI: 1.489–1.603	*N* = 1186 *p* = 0.0001 OR = 1.175 95% CI: 1.099–1.256	*N* = 1409
70–79 years	*N* = 2476 *p* = 0.0001 OR = 0.406 95% CI: 0.387–0.425	*N* = 3757 *p* = 0.0001 OR = 0.668 95% CI: 0.642–0.696	*N* = 6169 *p* = 0.0001 OR = 3.836 95% CI: 3.690–3.988	*N* = 559 *p* = 0.0001 OR = 0.683 95% CI: 0.624–0.747	*N* = 864
≥80 years	*N* = 1448 *p* = 0.0001 OR = 0.419 95% CI: 0.395–0.444	*N* = 1136 *p* = 0.0001 OR = 0.275 95% CI: 0.258–0.294	*N* = 4945 *p* = 0.0001 OR = 7.518 95% CI: 7.153–7.901	*N* = 149 *p* = 0.0001 OR = 0.295 95% CI: 0.250–0.347	*N* = 560
Others*	*N* = 04	*N* = 09	*N* = 00	*N* = 01	*N* = 34

* Category not defined.

## References

[1] Kuguyo O, Matimba A, Tsikai N (2017). Cervical cancer in Zimbabwe: a situation analysis. Pan Afr Med J.

[2] Hariri S, Dunne E, Saraiya M (2011). Human papillomavirus. VPD surveillance manual.

[3] de Peralta AM, Holaday B, Hadoto IM (2017). Cues to cervical cancer screening among U.S. Hispanic women. Hisp Heal Care Int.

[4] Sadalla JC, De Andrade JM, Genta MLND (2015). Cervical cancer: whats new?. Rev Assoc Med Bras.

[5] Rocha TAH, Da Silva NC, Thomaz EBAF (2017). Primary health care and cervical cancer mortality rates in Brazil: a longitudinal ecological study. J Ambul Care Manage.

[6] Galvin A, Delva F, Helmer C (2018). Sociodemographic, socioeconomic, and clinical determinants of survival in patients with cancer: a systematic review of the literature focused on the elderly. J Geriatr Oncol.

[7] Ministério da Saúde Secretaria Executiva DATASUS. Informações de Saúde.

[8] World Health Organization (2004). International Classification of Diseases.

[9] SIDRA, Instituto Brasileiro de geografia e Estatística (IBGE) Sistema IBGE de recuperação automática. https://sidra.ibge.gov.br/.

[10] Rosenberg PS, Check DP, Anderson WF (2014). A web tool for Age-period-cohort analysis of cancer incidence and mortality rates. Cancer Epidemiol Biomark Prev.

[11] Castle PE, Jeronimo J, Schiffman M (2006). Age-related changes of the cervix influence human papillomavirus type distribution. Cancer Res.

[12] Ho GY, Bierman R, Beardsley L (1998). Natural history of cervicovaginal papillomavirus infection in young women. N Engl J Med.

[13] Walboomers JM, Jacobs MV, Manos MM (1999). Human papillomavirus is a necessary cause of invasive cervical cancer worldwide. J Pathol.

[14] Bulk S, Visser O, Rozendaal L (2005). Cervical cancer in the Netherlands 1989–1998: decrease of squamous cell carcinoma in older women, increase of adenocarcinoma in younger women. Int J Cancer.

[15] Gao Y, Ma J, Gao F (2013). The evaluation of older patients with cervical cancer. Clin Interv Aging.

[16] Kong Y, Zong L, Yang J (2019). Cervical cancer in women aged 25 years or younger: a retrospective study. Cancer Manag Res.

[17] Herbert A, Singh N, Smith JA (2001). Adenocarcinoma of the uterine cervix compared with squamous cell carcinoma: a 12-year study in Southampton and South-west Hampshire. Cytopathology.

[18] Seoud M, Tjalma WA, Ronsse V (2011). Cervical adenocarcinoma: moving towards better prevention. Vaccine.

[19] Lau HY, Juang CM, Chen YJ (2009). Aggressive characteristics of cervical cancer in young women in Taiwan. Int J Gynaecol Obstet.

[20] Davy ML, Dodd TJ, Luke CG (2003). Cervical cancer: effect of glandular cell type on prognosis, treatment, and survival. Obstet Gynecol.

[21] Yang L, Jia X, Li N (2013). Comprehensive clinic-pathological characteristics of cervical cancer in southwestern China and the clinical significance of histological type and lymph node metastases in young patients. PLoS One.

[22] Lee KB, Lee JM, Park CY (2006). What is the difference between squamous cell carcinoma and adenocarcinoma of the cervix? A matched case-control study. Int J Gynecol Cancer.

[23] Vinh-Hung V, Bourgain C, Vlastos G (2007). Prognostic value of histopathology and trends in cervical cancer: a SEER population study. BMC Cancer.

[24] Rodrigues AN, de Melo AC, Alves FVG (2018). Lack of impact of race alone on cervical cancer survival in Brazil. Asian Pacific J Cancer Prev.

[25] Rauh-Hain JA, Clemmer JT, Bradford JS (2013). Racial disparities in cervical cancer survival over time. Cancer.

[26] Vale DB, Sauvaget C, Muwonge R (2016). Disparities in time trends of cervical cancer mortality rates in Brazil. Cancer Causes Control.

[27] Gao S, Manns BJ, Culleton BF (2008). Access to health care among status Aboriginal people with chronic kidney disease. CMAJ.

[28] Zuckerman S, Haley J, Roubideaux Y (2004). Health service access, use, and insurance coverage among American Indians/Alaska Natives and whites: what role does the Indian health service play?. Am J Public Health.

[29] Barcelos MRB, Lima RDCD, Tomasi E (2017). Quality of cervical cancer screening in Brazil: external assessment of the PMAQ. Rev Saude Publica.

[30] Instituto Brasileiro de Geografia e Estatística (2010). Censo demográfico.

[31] Silva NC, Rocha TAH, Rodrigues RB (2014). Equidade na atenção primária à saúde da mulher: uma análise do Brasil e suas regiões. Rev Baiana Saúde Pública.

[32] Santos RS, Melo ECP, Santos KM (2012). Análise espacial dos indicadores pactuados para o rastreamento do câncer do colo do útero no Brasil. Texto Contexto Enferm.

[33] Machida H, Eckhardt SE, Castaneda AV (2017). Single marital status and infectious mortality in women with cervical cancer in the United States. Int J Gynecol Cancer.

[34] Gomez SL, Hurley S, Canchola AJ (2016). Effects of marital status and economic resources on survival after cancer: a population-based study. Cancer.

[35] Ibrahimi S, Pinheiro PS (2017). The effect of marriage on stage at diagnosis and survival in women with cervical cancer. Psychooncology.

[36] Timoteo F, Korkes F, Baccaglini W (2020). Bladder cancer trends and mortality in the brazilian public health system. Int Braz J Urol.

